# Genetic association between LONG COVID and TMPRSS2 polymorphisms (rs12329760 and rs2070788) in Brazilian healthcare professionals

**DOI:** 10.3389/fcimb.2026.1731318

**Published:** 2026-06-15

**Authors:** Alysson Fellipe Costa Telles, Bearli Souza Menezes Junior, Cliomar Alves dos Santos, Ludmila Oliveira Carvalho Sena, Maria Rosa Melo Alves, Maria Luiza Aguiar Martins Moraes, Rosana Cipolotti

**Affiliations:** 1 Postgraduate Program in Health Sciences at the Federal University of Sergipe, Aracaju, Brazil; 2 Parreiras Horta Health Foundation, Aracaju, Brazil

**Keywords:** enzymological regulation of gene expression, genetic susceptibility, long Covid, occupational health, single nucleotide polymorphism

## Abstract

Long COVID syndrome has a multifactorial cause that is not fully understood and may be influenced by both external and intrinsic factors. In this context, a Genome-Wide Association Study (GWAS) was proposed to evaluate the association between Single Nucleotide Polymorphisms (SNPs) in TMPRSS2 (rs12329760 and rs2070788) and the occurrence of Long COVID in 363 Brazilian healthcare professionals, recruited using a non-probabilistic method. The study employed a self-report questionnaire to collect sociodemographic and clinical data from both the acute and chronic phases and also collected oral mucosa cells for genotypic analysis by qPCR using Taqman probes. The categorized information was analyzed using the PSPP software using Pearson’s chi-square test in three genetic statistical models: additive, dominant, and recessive. Assessing long COVID in general, only clinical variables such as increased susceptibility, presence of symptoms, and severity influenced the occurrence of the syndrome. When specifying the main reported symptom, brain fog, females and young adults (18 to 29 years old) are the most vulnerable, and rs2070788 in the recessive model proved to be relevant. This association is more evident when evaluating only the symptomatic group in the post-COVID period, as the additive model also influences the groups. rs12329760 showed no relevant influence on the groups. Therefore, this study provides unprecedented evidence of the association between brain fog and rs2070788, requiring case-control studies to better clarify how this association occurs.

## 1 Introduction

Long COVID has been widely discussed by the scientific community, following the definition provided by the World Health Organization (WHO), which characterizes it as the presence of symptoms resulting from COVID-19, lasting three months or more, without another diagnosis to justify these manifestations. The variety of symptoms and their frequency vary according to the population profile, with memory and concentration problems occupying a significant place in the reports of affected patients (Al-Oraibi et al., 2022; Kröönström et al., 2022; Cegolon et al., 2023; Fernández-de-las-Peñas et al., 2023a; Fernández-de-las-Peñas et al., 2023b; Huerne et al., 2023).

The causes of this heterogeneous profile are still unclear, and it is likely a multifactorial syndrome associated with environmental causes, such as prolonged exposure to the virus, and intrinsic host factors that may be modulating the process of symptom progression. Genetic alterations such as SNPs (single nucleotide polymorphisms) can affect the expression of proteins involved in the pathophysiology of this infection, acting as risk factors. Examples include rs12329760 and rs2070788 of the TMPRSS2 (Transmembrane Serine Protease 2) gene, an enzyme involved in the infectious process of viral entry into the host cell, and whose polymorphisms are associated with susceptibility and severity of the acute phase of the infection (Choudhary et al., 2021; Schönfelder et al., 2021; de Andrade et al., 2022).

rs12329760 is a missense SNP located in a coding region, promoting the translation of a different protein by replacing a valine with a methionine. Research worldwide has yielded controversial results regarding the influence of this polymorphism on the acute phase. However, when influential, the T allele appears to be associated with moderate TMPRSS2 catalytic activity due to the lower expression associated with this allele. rs2070788 is in a non-coding region and, to date, non-regulatory. However, the G allele is somehow associated with greater severity of COVID-19 cases due to increased protein expression. A possible linkage disequilibrium with other polymorphisms in the regulatory region may explain this association (Hou et al., 2020; Kehdy et al., 2021; Schönfelder et al., 2021; de Andrade et al., 2022).

Given the evidence of an association between these polymorphisms and the acute phase of COVID-19, a GWAS (Genotype Wide Association Study) study was proposed to evaluate the association between these two TMPRSS2 genetic polymorphisms and long COVID. To date, only one study in Spain has attempted to find this association, making this the second study worldwide with available results to use this data (Fernández-de-las-Peñas et al., 2022). Additionally, we wish to describe the sociodemographic and clinical profile of the population studied in the post-COVID period.

Despite all the knowledge produced about SNPs, the different ethnicities around the world and the diverse ancestry profiles demonstrate the importance of knowing the genetic profile linked to the people who inhabit the different continents, and the way in which genotypes impact the phenotypic characteristics manifested by each ethnic group (Choudhary et al., 2021; Galisa et al., 2021; Schönfelder et al., 2021). This study, conducted with a group of healthcare workers from a public hospital in Sergipe (the smallest state in Brazil, located in the Northeast region), is the first to address the association of these SNPs in this population, characterized by intense admixture and, consequently, great phenotypic diversity. The data referred to in this article are part of a macro-scale study, covering both the acute and post-COVID phases of these professionals.

## 2 Materials and methods

### 2.1 Recruitment of participants and collection of biological samples

Participant recruitment was conducted using a non-probabilistic convenience sampling method, providing easy access to hospital and administrative professionals at the University Hospital of Sergipe, which served as a referral center for patients infected with SARS-CoV-2 in the state of Sergipe between 2020 and 2021. As an eligibility criterion, all employees over 18 years of age were able to participate through a virtual and printed QR Code, which directed them to the Informed Consent Form (ICF) and the Clinical and Epidemiological Questionnaire (QCE) covering demographic and social data (gender, age, city of residence, and race), the acute phase of COVID-19 (number of positive tests, presence of symptoms, and severity), and the post-COVID profile (presence of symptoms, type of symptoms, and duration).

All responses were obtained through self-reporting, as severity stratification follows strict criteria. This study did not have access to participants’ medical records and reports to verify the number of positive tests, as COVID-19 diagnosis was decentralized throughout Brazil during the pandemic. The genetic evaluation used oral mucosa cells as a clinical sample, collected using swabs, following the guidelines of one of the studies that referenced this research (Araujo et al., 2021) and subsequently sent to the molecular biology laboratory of the Sergipe State Public Health Laboratory (Lacen-SE) at room temperature. The tubes were stored at 2 to 8 °C until genetic analysis began.

### 2.2 DNA extraction and qPCR

DNA was extracted using a silica column method using a commercial Qiagen^®^ kit, following the manufacturer’s instructions. A Thermo Scientific^®^ QuantStudio 5 thermal cycler processed the qPCR reactions, which followed the Taqman methodology, with Promega^®^ Master Mix and designed primers, labeled with probes, and distributed by Thermo Scientific^®^. **Table 1** below represents the nucleotide sequence of each target, as well as the polymorphisms (C/T for rs12329760 and A/G for rs2070788), with the probe that labels each allele.

**Table 1:** Characterization of the primer and probe sequences related to the molecular analysis by qPCR for the SNPs rs12329760 and rs2070788 of the TMPRSS2 enzyme.

SNP	Primer sequence	Probe	Assay code
rs12329760	CAGGACTTCCTCTGAGATGAGTACA**[C/T]**CTGAAGGATGAAGTTTGGTCCGTAG	C-VIC / T-FAM	C__25622353_20
rs2060788	GATTTGTTGTCTGTATGGCCTAGAC**[A/G]**CTTTTGAGAAGGATATAAACAATAT	A-VIC / G-FAM	C___2592038_1_

Source: https://www.thermofisher.com/order/genome-database/details/genotyping/C___2592038_1_; accessed on March 20, 2026

The standardized cycling for polymorphism amplification includes an enzymatic activation phase (60 °C for 1 minute) and the first denaturation phase (95 °C for 10 minutes). Subsequently, 40 amplification cycles were performed at 95 °C for 15 seconds and 60 °C for 1 minute, respectively, for denaturation and extension of the new strands. The final phase involved a final reaction at 60 °C for a final minute.

The results were expressed in an allelic discrimination graph, evaluated using three statistical genetic models, following the proposal of two Brazilian studies on genetic polymorphisms and COVID-19 (Tosta et al., 2023; de Almeida et al., 2024). The additive model assumes a linear increase based on the number of copies of the related allele present in the genotype; the dominant model assumes that one or two copies of the allele increase the risk; and the recessive model assumes that two copies of the allele are required to alter the risk associated with the trait/disease.

### 2.3 Statistical analysis

The QCE and genotyping data were categorized, and the PSPP (Portable Statistical Processing Program) software performed the statistical analyses, presenting the data in absolute and relative frequencies and using Pearson’s chi-square to establish associations between variables. The standardized confidence interval was set at 95% to indicate statistical significance (p < 0.05). QCE and genotyping data were categorized, and the PSPP (Portable Statistical Processing Program) software performed the statistical analyses, presenting the data in absolute and relative frequencies and using Pearson’s chi-square to establish associations between variables. The standardized confidence interval was set at 95% to indicate statistical significance (p < 0.05).

### 2.4 Ethical considerations

This research was submitted to Plataforma Brasil under CAAE number: 61307122.9.0000.5546 and approved for execution on July 11, 2022, by the Ethics Committee for Research involving Human Beings of the Federal University of Sergipe (CEP-UFS), following all the ethical precepts of said institution. All participants authorized participation through the TCLE, and the authors declare that there is no conflict of interest between the parties.

## 3 Results

After recruitment, 363 professionals scanned the QR Code to access and complete the QCE, and collected oral mucosa cells. Most participants were female (76%) and mainly adults (89.5% between 30 and 59 years old), with an average age of 41.4 years. Specifically, regarding the post-COVID period, 62.2% of participants reported experiencing some symptoms after acute SARS-CoV-2 infection, of which 31.7% no longer experienced these symptoms.

However, 30.6% reported still experiencing symptoms, characterizing long COVID syndrome, as the data were collected between 2024 and 2025, long after the acute phase. Of these, 11.6% had permanent clinical manifestations and 19% reported transient manifestations. Of the symptoms reported post-COVID, the main ones were problems with memory and concentration (31.6%), fatigue (26.8%), headache (18.6%), cough (15.3%), anxiety (13.3%), loss of smell and taste (12.4%), and muscle pain (11%). **Table 2** presents the data in absolute and relative frequencies (%).

**Table 2:** Characterization of participants by sex, age, and symptom profile post-COVID.

Variable	Categories	Abs	%
Sex	Male	87	24
	Female	276	76
Age	Young Adults (up to 29 years)	30	8.3
	Adults (30 to 59 years)	325	89.5
	Elderly (over 60 years)	8	2.2
Post-Covid Symptomatology	Presented symptoms in post-Covid	226	62.2
	Did not present symptoms post-COVID	137	37.8
Symptom Presentation Profile	Permanent, I feel it often.	42	11.6
	Transient, occasionally causing me discomfort.	69	19
	I had symptoms for a while, but now I don’t feel them anymore.	115	31.7
Main symptoms in Post-Covid	Problems with memory and concentration	112	31.6
	Fatigue	95	26.8
	Headache	66	18.6
	Cough	54	15.3
	Anxiety	47	13.3
	Loss of smell or taste	45	12.4
	Muscle pain (myalgia)	39	11

Table from author.

To find answers that explain the persistence of symptoms in the post-acute infection period, associations with sociodemographic variables (sex and age) and clinical variables (susceptibility, symptoms, and severity during acute COVID) were tested. The results show no statistically significant relationship between long COVID and the sociodemographic variables of this population, with all p-values greater than 0.05. However, they suggest a significant relationship with clinical variables, where participants with more detectable molecular tests, who had symptoms, and who progressed to more severe cases, were more likely to have prolonged COVID-related symptoms, without other underlying conditions to explain them.

Given the evidence that memory and concentration problems top the list of post-COVID symptoms in this population, consistent with the scientific literature, an association was proposed between this parameter and sociodemographic and clinical variables. A strong association was observed between sex (p=0.001) and age (p=0.006) and the presentation of these symptoms. Female gender appears to be a predictor of this condition, and after Bonferroni correction, young adults are more likely to have this condition. A logistic regression model was proposed, identifying an OR of 2.43 for having memory and concentration problems among females, and a 3.75 among young adults. Clinical variables behaved similarly to long COVID in general, with the number of positive tests, the presence/absence of symptoms, and severity influencing the chronicity of acute-phase symptoms. **Table 3** presents data related to both long COVID and memory and concentration problems.

**Table 3:** Association between social and clinical variables and reports of long COVID syndrome, and more specifically, with brain fog.

Sociodemographic and clinical variables of acute COVID		Didn’t have long COVID	Had long COVID	p value	Absence of brain fog	Presence of brain fog	p value
Sex	Male	38 (10.5%)	49 (13.5%)	0.076	72 (19.8%)	15 (4.1%)	0.001
	Female	95 (26.2%)	181 (49.9%)		178 (49%)	98 (27%)	
Age	Young Adults (up to 29 years)	14 (3.9%)	16 (4.4%)	0.19	27 (7.4%)	3 (0.8%)	0.006
	Adults (30 to 59 years)	118 (32.5%)	207 (57.0)		220 (60.6%)	105 (28.9%)	
	Elderly (Over 60 years)	1 (0.3%)	7 (1.9%)		3 (0.8%)	5 (1.4%)	
Number of positive tests	No positive tests	49 (13.5%)	16 (4.4%)	< 0.001	61 (16.8%)	4 (1.1%)	<0.001
	1 or 2 positive tests (average)	70 (19.3%)	129 (35.5%)		142 (39.1%)	57 (15.7%)	
	More than 3 positive tests	14 (3.9%)	85 (23.4%)		47 (12.9%)	52 (14.3%)	
Symptomatology Profile during Acute Covid	Asymptomatic	39 (10.7%)	3 (0.8%)	< 0.001	40 (11%)	2 (0.6%)	<0.001
	Symptomatic	94 (25.9%)	227 (62.5%)		210 (57.9%)	111 (30.6%)	
Severity	No symptoms	39 (10.7%)	4 (1.1%)	< 0.001	41 (11.3%)	2 (0.6%)	<0.001
	Mild symptoms	63 (17.4%)	97 (26.7%)		115 (31.7%)	45 (12.4%)	
	Severe symptoms	31 (8.5%)	129 (35.5%)		94 (25.9%)	66 (18.2%)	

Table from author.

Regarding the relationship between memory and concentration problems and the participants’ genotyping for the SNPs rs12329760 and rs2070788, in an innovative and pioneering way, significance was found for SNP 2070788 in the recessive model. By evaluating only patients with symptomatic post-COVID, to use a more uniform sample to confirm this association, it was possible to observe that both the additive and recessive models influence the manifestation of these symptoms. rs12329760 showed no association in any of the statistical models studied. **Tables 4** and **5** present the data and the association between the variables for the entire study group and only for participants with symptomatic post-COVID, respectively.

**Table 4:** Association between memory and concentration problems in Long Covid and genotyping for rs12329760 and rs2070788 for the entire sample.

SNP	Associated allele	Genetic model	Genotyping result	No reported brain fog (n=250)	Reported brain fog (n=113)	p value
rs12329760	C	Additive	CC	167 (46%)	75 (20.7)	0.93
			CT	72 (19.8%)	32 (8.8%)	
			TT	11 (3%)	6 (1.7%)	
		Dominant	TT	11 (3%)	6 (1.7%)	0.444
			CC+CT	239 (65.8%)	107 (29.5%)	
		Recessive	TT+CT	83 (22.9%)	38 (10.5%)	0.514
			CC	167 (46%)	75 (20.7%)	
rs2070788	A	Additive	AA	71 (19.6%)	46 (12.7%)	0.58
			AG	132 (36.4%)	47 (12.9%)	
			GG	47 (12.9%)	20 (5.5%)	
		Dominant	GG	47 (12.9%)	20 (5.5%)	0.463
			AA+AG	203 (55.9%)	93 (25.6%)	
		Recessive	AG+GG	179 (49.3%)	67 (18.5%)	0.014
			AA	71 (19.6%)	46 (12.7%)	

Table from author.

**Table 5:** Association between memory and concentration problems in long Covid and genotyping for rs12329760 and rs2070788 in patients with persistent post-Covid symptoms.

SNP	Associated allele	Genetic Model	Genotyping Result	No Reported Brain Fog (n=117)	Reported Brain Fog (n=113)	p value
rs12329760	C	Additive	CC	81 (35.2%)	75 (32.6%)	0.555
			CT	33 (14.3%)	32 (13.9%)	
			TT	3 (1.3%)	6 (2.6%)	
		Dominant	TT	3 (1.3%)	6 (2.6%)	0.233
			CC+CT	114 (49.6%)	107 (46.5%)	
		Recessive	TT+CT	36 (15.7%)	38 (16.5%)	0.373
			CC	81 (35.2%)	75 (32.6%)	
rs2070788	A	Additive	AA	29 (12.6%)	46 (20%)	0.032
			AG	65 (28.3%)	47 (20.4%)	
			GG	23 (10%)	20 (8.7%)	
		Dominant	GG	23 (10%)	20 (8.7%)	0.416
			AA+AG	94 (40.9%)	93 (40.4%)	
		Recessive	AG+GG	88 (38.3%)	67 (29.1%)	0.007
			AA	29 (12.6%)	46 (20%)	

Table from author.

Another noteworthy finding in post-COVID-19, due to its specificity during the infectious process and appearing in 12.4% of reports, is the loss of smell and taste. Along with flu-like symptoms, this clinical manifestation has helped characterize the profile of COVID-19 in healthcare settings worldwide. In this study, unprecedented, its persistence in post-COVID-19 syndrome also showed an association with rs2070788 in the recessive model, with a p-value of 0.028.

A binary logistic regression model was proposed to validate the associations found between brain fog and rs2070788 in the recessive model, where significance (p-value) and OR were calculated both for the overall sample (p=0.021; OR = 1.73) and for participants who reported symptoms related to long COVID-19 (p=0.011; OR = 2.08). The same cannot be said about the loss of smell and appetite during long COVID, since the logistic regression test did not present satisfactory results that supported the hypothesis of association with rs2070788 (p=0.112 and OR = 0.52), requiring additional analyses with larger samples, which could better demonstrate the strength of this relationship.

## 4 Discussion

Although participants were recruited using a non-probabilistic method, the data regarding gender agrees with the characterization of female participation described by the WHO (World Health Organization) and the IBGE (Brazilian Institute of Geography and Statistics) (Instituto Brasileiro de Geografia e Estatística (IBGE), 2021), which sees women contributing to approximately 70% of services provided in public and private hospitals. Other studies involving hospital workers, albeit using different sampling techniques, found similar frequencies regarding gender (Jacob et al., 2021; Caixeta et al., 2023; Cegolon et al., 2023; Cunha et al., 2023).

The variable “age” represented an average classified as productive, since the research addressed health workers, and the data are consistent with other similar data (Kröönström et al., 2022; Cegolon et al., 2023). They found similar averages when evaluating their complete sample stratified by gender. Despite the growing number of older adults in the labor market, the data proved to be conservative regarding age.

Regarding long COVID, the results agree with the clinical presentation of the syndrome in several scientific publications, presenting the same symptoms at very similar frequencies (Aiyegbusi et al., 2021; Davis et al., 2021; Fernández-de-las-Peñas et al., 2022; Wulf Hanson et al., 2022; Fernández-de-las-Peñas et al., 2023b; Huerne et al., 2023). One of them, in a multicenter format with health workers in Italy, brings the same group of symptoms as those found in this research, as the most prevalent (Cegolon et al., 2023). The sociodemographic variables did not show a statistical association with long COVID events, attributed once again to the non-probabilistic capture methodology, but the clinical data of the acute infection were significant, demonstrating the association between the acute and chronic phases of the infection.

The results available to the scientific community are quite controversial, which is justified by the multifactorial profile of long COVID. Some studies show a very strong association between chronic post-COVID symptoms and female gender and advanced age (Aiyegbusi et al., 2021; da Silva et al., 2023), while others associate it solely with the female gender, with the incidence being higher in younger individuals (35 to 50 years old) (Carod-Artal, 2021) and others where no association was found (Fernández-de-las-Peñas et al., 2022; Fernández-de-las-Peñas et al., 2023a). It is believed that the sampling profile used by these studies may be influencing these results, as well as other external factors that were not discussed by them.

The relationship between severity and susceptibility also yields conflicting results, highlighting the need for further studies of this syndrome. A review by UK researchers argues that symptoms persist regardless of the severity of the acute infection (Aiyegbusi et al., 2021). A study from England supports the hypothesis that severity is a factor associated with greater impairment post-COVID (Hampshire et al., 2024). Finally, a meta-analysis assumes that the number of published articles is not sufficient to conclude the influence of severity and long COVID, reinforcing the contribution of the data obtained with this study (Crivelli et al., 2022).

Long COVID was assessed in general terms, with the categorization of this variable considering the persistence of symptoms to the present day, whether transient or permanent. This assessment showed no statistical association with the genotyping results for the two SNPS, and although there was no significant relationship between the variables, there was a frequency distribution favorable to the presence of the T allele for rs12329760 and the G allele for rs2070788, similar to what was found in one of the references used for this study (Fernández-de-las-Peñas et al., 2022). A probabilistic model of group distribution would help to better visualize these relationships.

Memory and concentration problems stand out as the main symptoms reported by participants in this study. Scientific literature describes this phenomenon as “brain fog,” characterized primarily by confusion, difficulty concentrating, and short-term memory loss, related to complex and multifactorial causes (Davis et al., 2021; Fernández-de-las-Peñas et al., 2023a; Hampshire et al., 2024). Historically, other epidemics had already associated neurological complications and viral infections, such as the Spanish flu (1918–1921), and the Coronaviruses that preceded SARS-CoV-2, SARS-CoV in 2003, and MERS-CoV in 2012 (Crivelli et al., 2022).

Studies on this topic are not yet completely clear, but they suggest that possible causes for this symptom in long COVID include chronic and systemic inflammation caused by the virus, affecting the nervous system (neuroinflammation), the formation of micro clots that can obstruct brain capillaries, causing endothelial damage, and autoimmune conditions that can promote a continuous state of autoantibody production, which will constantly affect the nervous system (Crivelli et al., 2022; Fernández-de-las-Peñas et al., 2022; Hampshire et al., 2024).

According to the results of this study, there is an association between the variables sex and age and the manifestation of memory and concentration problems post-COVID, with female participants (p=0.001) and the young adult group (p=0.005 after Bonferroni correction) being the most likely to experience this symptom, supported by the results of logistic regression. The scientific community still debates these relationships extensively, with studies both favorable and unfavorable to the results found in this population, as well as the underlying causes. The profile of the female immune system, the greater predisposition to autoimmune diseases, and hormonal involvement are some of the arguments used to support the hypotheses on this topic (Aiyegbusi et al., 2021; Carod-Artal, 2021; Davis et al., 2021; Fernández-de-las-Peñas et al., 2023a).

Regarding genetic polymorphisms, rs12329760 does not appear to contribute to brain fog, but rs2070788 in the recessive model does (p=0.014), an unprecedented finding in scientific literature. Logistic regression shows that carriers of the G allele in this model have an odds ratio of 1.73 times more likely to experience memory and concentration problems. This result supports the relationship between this polymorphism and the severity of COVID-19 in the acute phase, in which the presence of the G allele influences more severe events, as well as contributing to the manifestation of memory and concentration problems in the long term (Cheng et al., 2015; de Andrade et al., 2022).

When evaluating only patients with post-COVID symptoms, this relationship becomes much closer, with the additive model (p=0.032) emerging as a predictor of brain fog on the GG > AG > AA scale, alongside the recessive model, which strengthens its association with a p-value of 0.007. Logistic regression also highlights a strengthening of the relationship, with an OR of 2.08. It is possible that, given the association of the G allele with higher enzyme expression, this polymorphism is also helping to modulate cases of long COVID, with chronic symptoms in other systems. In the case of brain fog, it can cause damage to the nervous system.

According to GTEx, the lungs naturally have a moderate expression of TMPRSS2 but are not the tissue with the highest absolute expression of this enzyme. The prostate and gastrointestinal tract appear to express TMPRSS2 the most, but they do not represent a direct entry point for this virus, unlike the respiratory tract, which becomes the main route of transmission (Irham et al., 2020; Pandey et al., 2022). Given the increased expression caused by the G allele, evidenced by this study and other GWAS, the chances of increased TMPRSS2 in other systems may serve as a hypothesis to justify the appearance of other symptoms, such as those in the nervous system, which originally present low expression of this protein.

Because this is a complex and multifactorial syndrome, this study was only able to demonstrate a significant association between the variables. Only one study in Spain investigated this relationship and was unsuccessful in establishing a correlation (Fernández-de-las-Peñas et al., 2022), most likely due to the sample profile, which included previously hospitalized patients with a lower average age than this study, and a smaller sample size (293). Despite this, the Spanish study better represented the gender, with 49.5% women and 50.5% men. A future evaluation proposal, with a case-control study with people who had acute COVID-19 and did not develop any post-COVID symptoms, composing the control group, will be necessary to confirm this unprecedented association, as well as to try to better elucidate the cause-and-effect relationship, similar to what was proposed in a Brazilian study carried out in Belém/PA (da Silva et al., 2023). Probabilistic selection methods should adjust the results and further highlight these relationships.

## 5 Conclusion

An association between sex and age (women in the young adult group) and memory and concentration problems in long COVID was demonstrated, and for the first time, with the rs2070788 of the TMPRSS2 enzyme in the recessive model, demonstrating the importance of this polymorphism in epidemiological research, as it is a predictor of the severity and chronicity of these symptoms. Further studies are needed to clarify this influence, but there may be a relationship with the greater expression of TMPRSS2 that the G allele promotes in the host.

Because this is a complex and multifactorial syndrome, the statistics applied to these data were only able to demonstrate a significant association between the variables. A future evaluation proposal, with a case-control study of people who had acute COVID-19 and did not develop any post-COVID symptoms, will be necessary to confirm this unprecedented association, as well as to further elucidate the cause-and-effect relationship. Probabilistic selection methods should adjust the results and further demonstrate these relationships. It is worth noting that, although the sample is in Hardy-Weinberg equilibrium, genetic evaluations should not be generalized to any population, and the ancestry profile of the population should always be considered for better associations.

## Data Availability

The clinical and epidemiological questionnaire is available on the Zenodo platform, in both Portuguese and English versions, as well as the raw data obtained from the participants’ responses to this research. The web address for viewing on the platform is https://doi.org/10.5281/zenodo.18779029. The participants’ sensitive data was preserved, in compliance with the confidentiality terms and Brazilian data protection legislation (Law No. 13,709/2018 - LGPD).
